# Epithelial Label-Retaining Cells Are Absent during Tooth Cycling in *Salmo salar* and *Polypterus senegalus*

**DOI:** 10.1371/journal.pone.0152870

**Published:** 2016-04-06

**Authors:** Sam Vandenplas, Maxime Willems, P. Eckhard Witten, Tom Hansen, Per Gunnar Fjelldal, Ann Huysseune

**Affiliations:** 1 Evolutionary Developmental Biology, Ghent University, Ghent, Belgium; 2 Pharmaceutical technology, Ghent University, Ghent, Belgium; 3 Institute of Marine Research (IMR), Matre Research Station, Matredal, Norway; Team 'Evolution of Vertebrate Dentition', FRANCE

## Abstract

The Atlantic salmon (*Salmo salar*) and African bichir (*Polypterus senegalus*) are both actinopterygian fish species that continuously replace their teeth without the involvement of a successional dental lamina. Instead, they share the presence of a middle dental epithelium: an epithelial tier enclosed by inner and outer dental epithelium. It has been hypothesized that this tier could functionally substitute for a successional dental lamina and might be a potential niche to house epithelial stem cells involved in tooth cycling. Therefore, in this study we performed a BrdU pulse chase experiment on both species to (1) determine the localization and extent of proliferating cells in the dental epithelial layers, (2) describe cell dynamics and (3) investigate if label-retaining cells are present, suggestive for the putative presence of stem cells. Cells proliferate in the middle dental epithelium, outer dental epithelium and cervical loop at the lingual side of the dental organ to form a new tooth germ. Using long chase times, both in *S*. *salar* (eight weeks) and *P*. *senegalus* (eight weeks and twelve weeks), we could not reveal the presence of label-retaining cells in the dental organ. Immunostaining of *P*. *senegalus* dental organs for the transcription factor Sox2, often used as a stem cell marker, labelled cells in the zone of outer dental epithelium which grades into the oral epithelium (ODE transition zone) and the inner dental epithelium of a successor only. The location of Sox2 distribution does not provide evidence for epithelial stem cells in the dental organ and, more specifically, in the middle dental epithelium. Comparison of *S*. *salar* and *P*. *senegalus* reveals shared traits in tooth cycling and thus advances our understanding of the developmental mechanism that ensures lifelong replacement.

## Introduction

The enthralling ability to continuously replace teeth throughout life has fascinated scientists for decades. This ability is maintained in almost all non-mammalian vertebrates and is a topic of growing interest. Research has recently focused on the putative involvement of stem cells in continuous tooth replacement in a wide range of species: lesser spotted catshark (*Scyliorhinus canicula*) [1–3]; African bichir (*Polypterus senegalus*) [4]; rainbow trout (*Oncorhynchus mykiss*) [5–7]; Atlantic salmon (*Salmo salar*) [8,9]; zebrafish (*Danio rerio*) [10–12]; medaka (*Oryzias latipes*) [13]; leopard gecko (*Eublepharis macularius*) [14,15] and corn snake (*Pantherophis guttatus*) [16]. These studies reveal considerable interspecific variation in morphological features, organization and patterning of the dentition; however almost all species have teeth that are organized in tooth families, i.e. one functional tooth and all of its successors [17]. Reif [17,18] proposed that the dentition of all jaw bearing vertebrates is formed by a dental lamina, i.e. a deep epidermal invagination in which successor teeth develop before the old teeth are shed or resorbed. This dental lamina can differ in morphological or spatio-temporal characteristics and was categorized by Reif [17] either as (1) continuous or discontinuous or (2) as permanent or non-permanent. Examples for (1) are the continuous dental lamina in *S*. *canicula* [17] and the discontinuous dental lamina in the cichlid *Hemichromis bimaculatus* [19,20]. Examples for (2) are the permanent dental lamina in *Syciopterus japonicus* [21] and the non-permanent dental lamina in *D*. *rerio* [22]). Yet, studies in *O*. *mykiss* [7,23], *S*. *salar* [8] (both closely related basal protacanthopterygian teleosts), and more recently *P*. *senegalus* [4] (a living representative of a basal clade within the actinopterygians), revealed the absence of a dental lamina as defined by Reif [17]. In these species, successor teeth develop directly from the lingual outer dental epithelium covering the predecessor teeth. Here, an epithelial tier is positioned between the inner dental epithelium (IDE) and outer dental epithelium (ODE) [8]. The latter authors coined the term middle dental epithelium (MDE) for this tier, and hypothesized that it could functionally substitute for a dental lamina by supplying the outer dental epithelium with cells before its differentiation into a placode.

Given the suggested possible involvement of epithelial stem cells in continuous tooth replacement [10], the dental lamina, or the MDE for that matter, has been considered the obvious potential source for such stem cells [3,8,22]. However, until now, little evidence has been found for stem cell involvement in tooth cycling of actinopterygians.

Stem cells are mainly characterized by their ability for self-renewal, i.e. they have the capacity to undergo numerous cell cycles, and produce progeny, while maintaining their undifferentiated state, even after a long inactive period [24]. Dependent on stem cell potency, their progeny gives rise to various differentiated cells either directly, or indirectly via transient amplifying cells. Stem cells reside in a stem cell niche, which can be defined as a strictly regulated microenvironment that maintains the stem cells and their function [25]. Because of their undifferentiated state, stem cells are difficult to identify [26]. Therefore many studies have to rely on indirect evidence to locate putative stem cells, such as slow cell cycle or the expression of particular transcription factors, e.g., SRY (sex determining region Y)-box 2 (*sox2*). This transcription factor maintains the pluripotency of early embryonic cells and regulates the formation of several epithelia during foetal development [27–30]. Arnold and colleagues [31] showed *sox2* expression in numerous adult endodermal and ectodermal stem cell compartments. In the mouse incisor, *sox2* expression has been observed in the labial cervical loop, a site known to contain epithelial stem cells [32]. Recently, *sox2* expression has been reported from the dental lamina giving rise to successional teeth in mammals (which display maximally only one round of tooth replacement), as well as in reptiles (characterized by continuous tooth replacement) [33]. Furthermore, Gaete and Tucker [16] described the presence of *sox2* transcripts in the dental lamina of corn snake (*Pantherophis guttatus)* dental slice cultures and Abduweli and colleagues [13] demonstrated *sox2* expression in the posterior end of a tooth family in the medaka (*O*. *latipes)*, a teleost fish.

Slow cycling cells can be detected when performing a pulse-chase experiment with, e.g., BrdU (5-Bromo-2’-deoxyuridine), a thymidine analogue that is incorporated in the nucleus of cells in the S-phase of the cell cycle. Over time, the BrdU in proliferating cells is diluted (yielding fragmented label in their nucleus), except in those that are slow-cycling, and are therefore termed label-retaining cells, LRCs. Various studies have used this method to collect evidence for the location of both known and unknown stem cell niches. LRCs have been shown in dental systems such as in the continuously growing rodent incisors and in the molars [34–37], in *E*. *macularius* [14] and in *O*. *latipes* [13]. However, a recent study failed to show LRCs in *P*. *senegalus* [4]. Whether this failure is related to the absence of a dental lamina is not known but can be tested using another species where teeth derive directly from the dental organ of the predecessor, such as the salmonid *S*. *salar*.

The dentition of salmonids, in particular that of *O*. *mykiss* and *S*. *salar*, has been well studied in terms of patterning of first and later generation teeth [5,6,9,38–41], tooth ontogeny and chronology of tooth development [23,42] and gene expression patterns [7,43,44]. In addition, Berkovitz and Moore [6] found that the length of the replacement cycle in *O*. *mykiss* varies between eight and thirteen weeks depending on the fish length (such data are not available for *S*. *salar* and *P*. *senegalus)*. However, whether the MDE functionally substitutes for a dental lamina and is a source of epithelial stem cells, as discussed by Huysseune and Witten [8], has not been investigated. Here, we focus on the lower jaw dentition of *S*. *salar* to test this hypothesis. In particular, we want to (1) determine the localization and extent of proliferating cells in the dental epithelial layers, (2) describe cell dynamics through a BrdU pulse-chase experiment and (3) investigate if label-retaining cells are present, suggestive for the putative presence of stem cells. Furthermore, (4) we want to expand our data set on *P*. *senegalus* [4] by using long BrdU chase times. Finally, (5) we determine the distribution of the transcription factor Sox2 within the dental organ. Comparison of both species allows us to assess whether they share proliferation characteristics. Given the phylogenetic position of *S*. *salar* as a basal protacanthoperygian, and *P*. *senegalus* as one of the most basal extant actinopterygians, our results can shed light on the developmental mechanism that ensures lifelong replacement in actinopterygian fishes.

## Materials and Methods

### Animals

A total of 48 *S*. *salar* (parr stage of the life cycle) with an average weight of 10 g and average fork length of 9 cm (i.e. length of a fish measured from the tip of the snout to the posterior end of the middle caudal rays [8]) were used for BrdU administration. They were obtained from the Havforskningsinstituttet (Institute of Marine Research, IMR, Matre, Norway) and had been reared in freshwater at a temperature of 13°C. Six *P*. *senegalus* with an average body length of 11.1 cm were used for BrdU administration and housed and handled as described in a previous study [4].

### BrdU administration

The *S*. *salar* specimens were injected intraperitoneally with 100 μl (10 μl/g body weight) of a solution containing 10mg/ml 5-Bromo-2’-deoxyuridine (32.6 mM) (BrdU, Sigma-Aldrich) in 0.1 M phosphate-buffered saline (PBS, pH 7.2). The injection was repeated 5 times every 12 hours for each fish, in an attempt to also label cycling cells which were not in the S-phase of their cell cycle during the first injection. Prior to manipulation, all fish were anaesthetized (Finquel, 100mg/L). Sampling of the fish occurred at different time points: 4 hours past the last BrdU injection as a pulse, and one (T1), two (T2), four (T3) and eight weeks (T4) after the last BrdU injection as chase time. These chase times were chosen based on wax imprint experiments conducted on a closely related salmonid i.e. *O*. *mykiss*, where the tooth life cycle of a specimen with a body length of 15 cm, was 16 weeks [23].

BrdU was administered to *P*. *senegalus* exactly as in *S*. *salar* and described in Vandenplas et al. [4]. Sampling occurred at six weeks (T5), eight weeks (T6) and twelve (T7) weeks after the final pulse. All fish were handled with permission of the ethical commission of the Faculty of Science of Ghent University (EC2011-036), which specifically approved the whole study.

### Tissue fixation, decalcification and sectioning

The *S*. *salar* were anaesthetized (Finquel, 100mg/L) and killed by decapitation. Lower jaws were dissected and fixed in 4% paraformaldehyde (PFA, pH 7.2) at 4°C for 48 hrs. The jaws were rinsed three times in 0.1 M PBS and decalcified for at least 3 weeks in Morse solution: 22.5% formic acid, 10% sodium citrate (Morse, 1945) prior to embedding in paraffin. Blocks were sectioned sagittally or frontally (coronally) at 5 μm (Prosan Microm HM360). The *P*. *senegalus* were anaesthetized, fixed, decalcified and sectioned as described in Vandenplas et al. [4]

### Immunohistochemistry

Immunohistological staining for BrdU on the paraffin sections was performed as described in [45] and [4]: rehydration through a decreasing ethanol series, chromatin precipitation in hydrochloric acid, block in 3% BSA/ 1% milk powder, exposure to primary antibody (monoclonal anti-BrdU antibody produced in Rat, ab6326, Abcam) and secondary antibody (polyclonal anti-rat Alexafluor 488, a11006, Invitrogen), DAPI counterstaining (1μl/ml) and mounting with Vectashield (Vector laboratories Inc., Burlingame, USA). The detection of Sox2 was done by using a heat-mediated antigen retrieval step in citric acid (pH 6) for 20 minutes at 95°C, and a step in blocking solution containing 2% BSA and 10% goat serum. We used an anti-Sox2 primary antibody produced in rabbit (ab97959, Abcam) in a concentration of 1/500 at 4°C overnight, and an anti-rabbit secondary antibody Dylight 488 (ab98507, Abcam) at a concentration of 1/300 for one hour. The sections were counterstained with DAPI (1μl/ml distilled water) and mounted with Vectashield (Vector laboratories). Immunofluorescence was visualized on a NIKON eclips TE2000-S confocal laser-scanning microscope. Adobe Illustrator CS5, Adobe Photoshop CS5 and Fiji were used to process bright field and fluorescent images of the immunostained sections. The number of tooth families examined for immunohistochemistry (n) is indicated throughout the results and representative sections were chosen for the figure plates.

### Three-dimensional reconstruction

Histological sections of *S*. *salar* stained with toluidine blue were from material described in [8]. Photographs of these sections were taken on a Zeiss Axio Imager 0.Z.1 equipped with a Zeiss Axiocam MRc camera. In order to understand the spatial relationships between the functional tooth (predecessor) and the developing replacement tooth (successor), and to identify the potential localization of a stem cell niche we made three-dimensional reconstructions of the dental organ of a single tooth family by manually aligning pictures from 28 consecutive sections and generating surfaces in the Amira 3D 5.6 software.

### Quantification

The ratio of BrdU^+^ to DAPI^+^ cells was determined in different layers (outer dental epithelium, ODE; inner dental epithelium, IDE, middle dental epithelium, MDE; dental papilla, DP and pulp cavity, PC) of a tooth family by counting BrdU and DAPI labelled cells in successive sections of 5μm (T0: 5 sections, T2: 5 sections). Double counting of nuclei could not be excluded; therefore, the ratio could be a slight underestimate. Since only one sample was counted for each time point, no statistical analysis was performed on these results. However, given the high number of cells counted, it can be used as a proxy to detect preliminary trends in cell dynamics. All cell counting was performed manually and processed with Fiji software.

## Results

**Fig 1:**
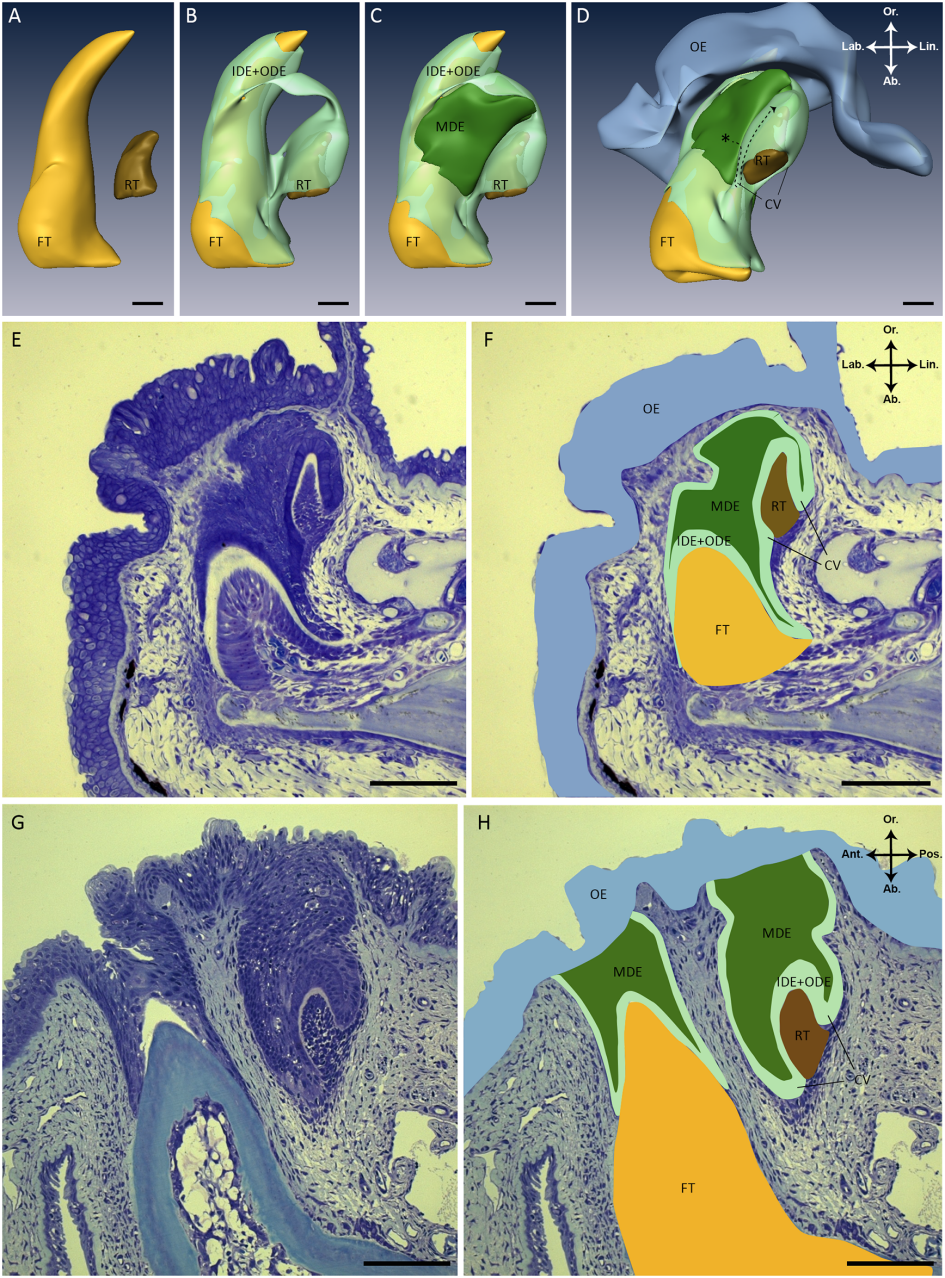
Structure of the dental organ in *S*. *salar*. (A-D) Three-dimensional reconstructions of a tooth family on the lower jaw in a juvenile *S*. *salar* (fork length: 4.84 cm). To simplify, the inner dental epithelium (IDE) of the functional tooth and of its successor were combined with the outer dental epithelium (ODE) into one layer (Fig 1B to 1D, light green). Please note that the smoothened surface facing the observer represents the edge of the 3D reconstruction and does not indicate that the MDE lies superficially. Instead, the MDE is covered by IDE+ODE outside the area of reconstruction. (D) The same three-dimensional reconstruction as in (A-C) but slightly tilted. The hypothesis to be tested is that stem cells (asterisk) reside in the middle dental epithelium (MDE), and give rise to transit amplifying cells, populating the cervical loop to eventually become differentiated ameloblasts (dashed arrow). (E) Sagittal histological section (2μm) through a tooth family of a juvenile *S*. *salar* (fork length: 4.84 cm) stained with toluidine blue. These serial sections are used to generate the three-dimensional reconstructions in (A-D). (F) Same histological section as in (E) with different structures overlaid in the colors used in the three-dimensional reconstructions (A-D). (G) Transverse histological section (2μm) through two adjacent tooth families of juvenile *S*. *salar* (fork length: 10 cm). In one tooth family the replacement tooth is not visible and lies behind the plane of view, while in the other tooth family the functional tooth is not visible because it lies above the plane of view. (H) Same histological section as in (G) with different structures overlaid in the colors used in the three-dimensional reconstructions (A-D). Abbreviations: Ab: aboral; Ant: anterior; CV: cervical loop; FT: functional tooth; IDE: inner dental epithelium; Lab: labial; Lin: lingual; MDE: middle dental epithelium; OE: oral epithelium; ODE: outer dental epithelium; Or: oral; Pos.: posterior; RT: replacement tooth; asterisk: hypothetical stem cell niche; color codes: yellow (FT), brown (RT), light green (IDE+ODE), dark green (MDE), blue (OE); scale bars: 80 μm.

The dental organ in *S*. *salar* is a spatially complex structure that encloses two tooth members. The 3D reconstruction shows a young functional tooth associated with a successor tooth in an early cytodifferentiation stage of development (Fig 1A to 1D). The dental organ is composed of the inner dental epithelium (IDE) of the functional tooth, the IDE of its successor, a middle dental epithelium (MDE) and the outer dental epithelium (ODE). The IDE is a single cell layer, which covers the dental papilla of the replacement tooth, as well as the functional tooth, except at its tip. Lingually the IDE of the successor grades via the cervical loop into the ODE (Fig 1D). The ODE represents the outer cell layer of the dental organ and connects without clear boundary to the basal layer of the oral epithelium. The MDE is enclosed by IDE and ODE at the lingual side of the functional tooth (Fig 1C and 1D, dark green). This tier has an irregular shape with the thickest part situated between the functional tooth and the tip of the successor. Towards the cervical loop of the successor tooth, the MDE becomes more narrow. The MDE extends as a single cell layer between the IDE and ODE of the successor.

### Cells proliferate at the lingual side of the dental organ to form a new tooth germ

We next used the spatial insights obtained through 3D reconstruction to assess the orientation of individual histological sections through the different tooth families in our pulse & chase experiment in *S*. *salar*. In particular, we made sure to study the label in those sections representing the most advanced stage of development of the tooth germ, rather than in sections hitting its less advanced periphery. Note that in all further descriptions, we use ‘dental organ’ to denote the epithelial structure common to predecessor and successor, and thus including the IDE and ODE of the predecessor, the MDE, and the IDE and ODE of the successor.

**Fig 2:**
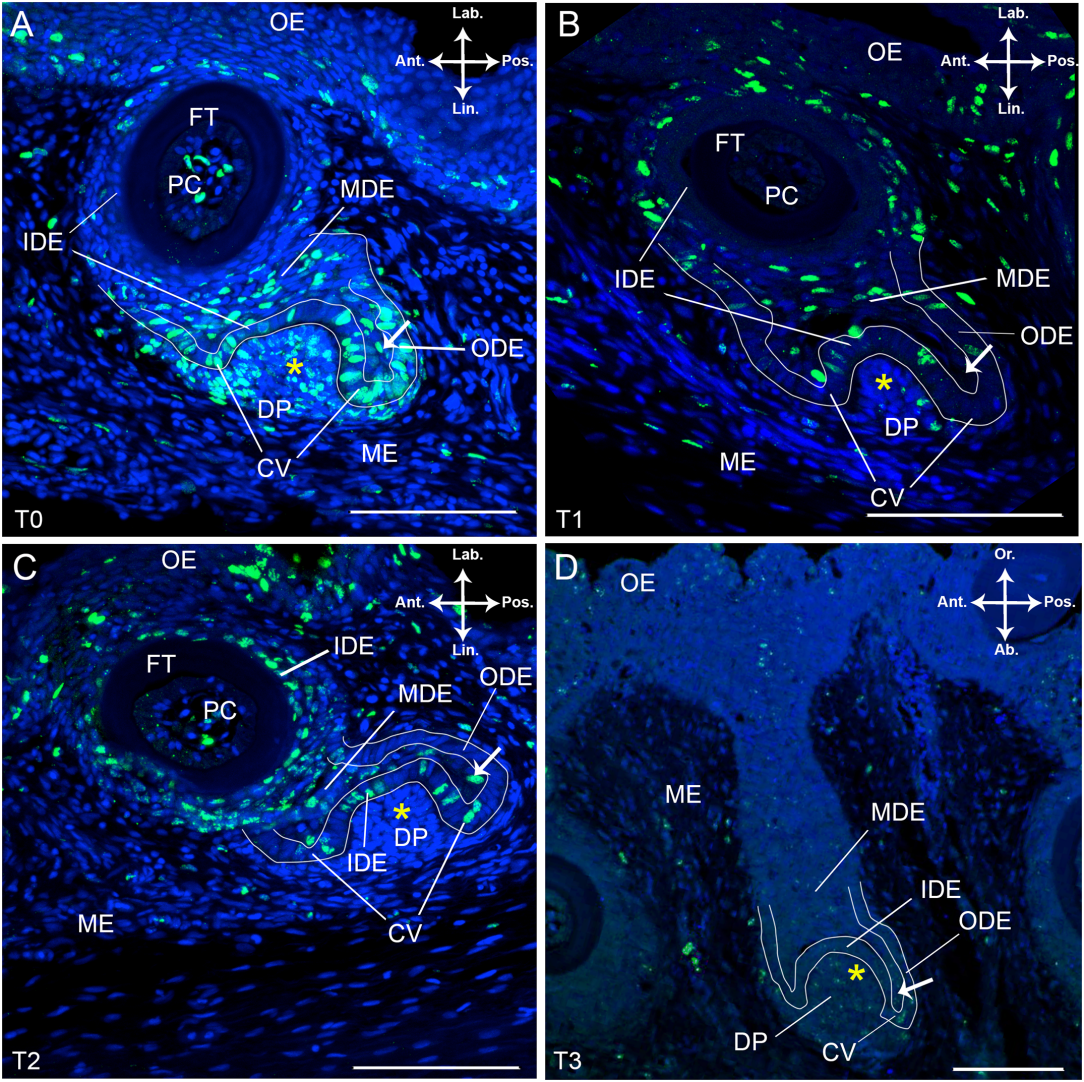
BrdU labelled tooth families in *S*. *salar* chased over different periods. Immunohistological staining of frontal (A-C) and sagittal (D) sections through a tooth family on the lower jaw of *S*. *salar* with BrdU labelled cells in green and DAPI counterstain in blue. The tooth families presented are in a similar state of development: a young functional tooth and its successor in morphogenesis stage. (A) Pulsed specimen, T0; (B) Chase time T1, one week after BrdU administration; (C) Chase time T2, two weeks after BrdU administration and (D) Chase time T3, four weeks after BrdU administration. The white arrow indicates single cells within the middle dental epithelium (MDE) enclosed by the IDE, cervical loop and ODE of the replacement tooth. Abbreviations: Ab: aboral; Ant: anterior; CV: cervical loop; DP: dental papilla; FT: functional tooth; IDE: inner dental epithelium; Lab: labial; Lin: lingual; MDE: middle dental epithelium; ME: mesenchyme; OE: oral epithelium; ODE: outer dental epithelium; Or: oral; PC: pulp cavity; Pos: posterior; yellow asterisk: replacement tooth; scale bars: 100 μm.

**Fig 3:**
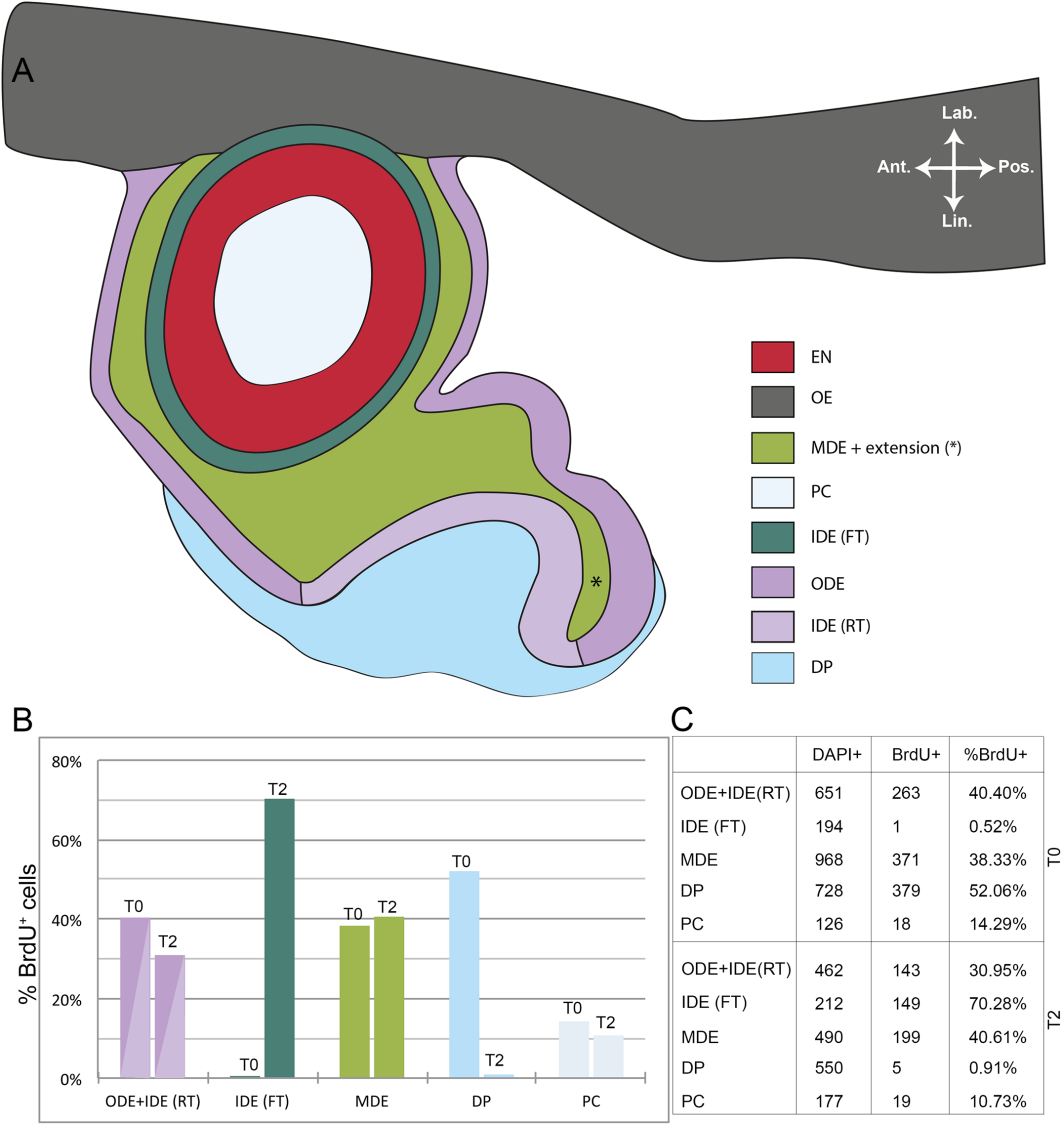
Ratio of BrdU^+^ to DAPI^+^ cells after pulse and after two weeks of chase time in *S*. *salar*. (A) Schematic representation of the different layers in a tooth family containing two members: a young functional tooth and a successor in morphogenesis stage. (B) Graphical representation of the ratio of BrdU^+^ to DAPI^+^ cells in the different layers as depicted in (A). (C) Table showing results from cell counting in the different layers. The data were acquired from five consecutive sections in both T0 (pulsed specimen) and T2 (two weeks after BrdU administration). Abbreviations: Ant: anterior; DP: dental papilla; EN: enameloid; FT: functional tooth; IDE: inner dental epithelium; Lab: labial; Lin: lingual; MDE: middle dental epithelium; OE: oral epithelium; ODE: outer dental epithelium; PC: pulp cavity; Pos: posterior; RT: replacement tooth.

Sagittal and frontal sections through the lower jaw of *S*. *salar* that received a pulse (indicated as T0, see Material and Methods) show BrdU labelled nuclei in the dental organ, the oral epithelium and the mesenchyme (Fig 2A, see Fig 3 for identification of the different cell layers). The distribution of BrdU^+^ cells within the dental organ appeared to be dependent on the developmental stage of the members of the tooth family (n = 5). Thus, a tooth family containing a replacement tooth in morphogenesis stage shows BrdU labelled nuclei in the ODE at the lingual and posterior side of the dental organ (n = 3, Fig 2A). Labially, this layer merges into the basal layer of the oral epithelium (OE), has no BrdU labelled cells and is further referred to as the ODE transition zone. The IDE of the replacement tooth contains BrdU labelled nuclei close to the cervical loop; towards the tooth tip labelled cells are only occasionally seen. In the IDE of the predecessor BrdU^+^ cells are absent (Fig 2A). In the cervical loop of the successor, which is the transition zone between ODE and IDE, all cells are BrdU-positive.

The MDE has many BrdU^+^ cells, distributed throughout the entire tier. Different from the ODE and IDE, the cell nuclei are flattened (Fig 2A). A few cells are enclosed by the cervical loop at the posterior side of the replacement tooth and are labelled for BrdU (Fig 2A, arrow). These cells are also part of the MDE. These flattened cells are orientated perpendicular to the adjacent cells in the IDE or ODE (Fig 2A). Given its association with a successor in morphogenesis stage, the functional tooth is still young. Its pulp cavity contains few BrdU^+^ cells, restricted to the centre region, and more frequent at the level of the base of the tooth than at the tip. Almost all mesenchymal cells that constitute the dental papilla of the successor are BrdU^+^ (Fig 2A). Some of these cells have a slightly fragmented BrdU label when compared to the intact label in cells of the IDE, ODE and MDE, indicating they divided already during the pulse time. Furthermore, this condensation of mesenchymal cells extends beyond the boundaries delimited by the IDE of the successor, as it continues lingual to the cervical loop and along the ODE. Likewise, most of these cells are labelled. Elsewhere in the mesenchyme surrounding the dental organ, BrdU labelled cells are only occasionally found. In the oral epithelium, BrdU^+^ cells are restricted to the basal two to three tiers.

Next, we compared the pattern of BrdU labelling over different chase times in tooth families displaying a nearly similar state of development as described above, i.e. tooth families with a young functional tooth and a successor in early morphogenesis stage. After one week of chase time (T1), the ODE contains BrdU labelled cells predominantly around the functional tooth, both on posterior and anterior side, and much less so around the replacement tooth (n = 5, Fig 2B). Only a few BrdU^+^ cells with fragmented label are present in the cervical loop of the replacement tooth (data not shown). The IDE of the functional tooth is BrdU^-^ and the IDE of the successor shows a small number of BrdU labelled cells, mostly with a fragmented label (Fig 2B). The MDE displays many BrdU^+^ cells, either with intact or fragmented label (Fig 2B). The cells within the extension of the MDE, enclosed by the cervical loop of the replacement tooth, show no BrdU label. The pulp cavity of the functional tooth has only a few BrdU^+^ cells located in the centre and aborally. The dental papilla of the replacement tooth (including the mesenchymal condensation extending lingual to the cervical loop and ODE) displays a few BrdU^+^ cells, albeit with a fragmented BrdU label (Fig 2B).

After two weeks of chase time (T2), only a few cells in the ODE around the functional tooth are BrdU-positive, with most of the nuclei having a fragmented label (n = 8, Fig 2C). At its base, the functional tooth shows a high number of cells with non-fragmented BrdU label in the IDE. In contrast, labelled cells are absent from this layer at the tooth tip. In the replacement tooth, many cells in the IDE display BrdU label in the nucleus, albeit fragmented (Fig 2C). In the MDE BrdU^+^ cells show a distinct pattern of labelling. Cells in close proximity to the functional tooth have fragmented BrdU label while cells close to the IDE of the successor tooth possess non-fragmented BrdU label (Fig 2C). A BrdU^+^ cell with intact label is observed in the MDE extension enclosed by the cervical loop (Fig 2C, arrow). The pulp cavity of the functional tooth shows BrdU-labelled cells at its base but none at its tip. BrdU^+^ cells in the dental papilla of the replacement tooth are restricted to some faintly labelled cells with only small stained fragments in their nucleus.

After four weeks of chase time (T3), only few BrdU^+^ cells persist in the dental organ (n = 5, Fig 2D). In the MDE and its extension within the cervical loop of the successor, faint and fragmented BrdU label is found in a limited number of cell nuclei (Fig 2D). The dental papilla of the replacement tooth likewise displays fragmented BrdU label. However, non-fragmented BrdU label is present in mesenchymal cells surrounding the dental organ at its aboral, anterior and posterior side. The oral epithelium shows many nuclei with fragmented and non-fragmented BrdU label in its outermost tiers. Occasionally, BrdU^+^ cells are present in the basal layer of the oral epithelium and in the ODE transition zone between oral epithelium and ODE (Fig 2D).

We calculated the ratio of BrdU^+^ to DAPI^+^ cells in the different parts of epithelium and mesenchyme from one tooth family in a similar developmental stage between pulse time T0 and chase time T2 (Fig 3A to 3C). To this end, BrdU^+^ cells were counted through five consecutive sections. No distinction was made between cells with fragmented and non-fragmented BrdU label. The number of BrdU^+^ cells in the ODE and IDE of the replacement tooth was lower after two weeks of chase time (T2) compared to the pulse (T0). The IDE of the functional tooth showed almost no BrdU^+^ cells in T0 while the percentage of BrdU^+^ cells in T2 was as high as around 70% (Fig 3B and 3C). The opposite is observed in the dental papilla of the successor: while a large fraction of cells was BrdU^+^ at T0, nearly all BrdU label was lost after two weeks of chase time. The ratio of BrdU^+^ to DAPI^+^ cells in the MDE was rather similar between T0 and T2 and fairly high (around 38–40%) (Fig 3C). Finally, the percentage of BrdU-labelled cells in the pulp cavity of the functional tooth had slightly decreased after two weeks of chase time (Fig 3B and 3C).

To summarize the above observations, the MDE, ODE and cervical loop are highly proliferative. Interestingly, the extension of the MDE, enclosed by the cervical loop of the developing tooth germ, contains some BrdU-labelled cells. Over time, the BrdU labelled cells show increasingly fragmented label in the nucleus leading to a complete failure to find any brightly stained cells with intact label in the dental organ after four weeks of chase time. Nonetheless, in the oral epithelium cells with non-fragmented label persist until this moment.

### Cells with intact BrdU label are absent in the dental organ of *Salmo salar* after eight weeks chase time

**Fig 4:**
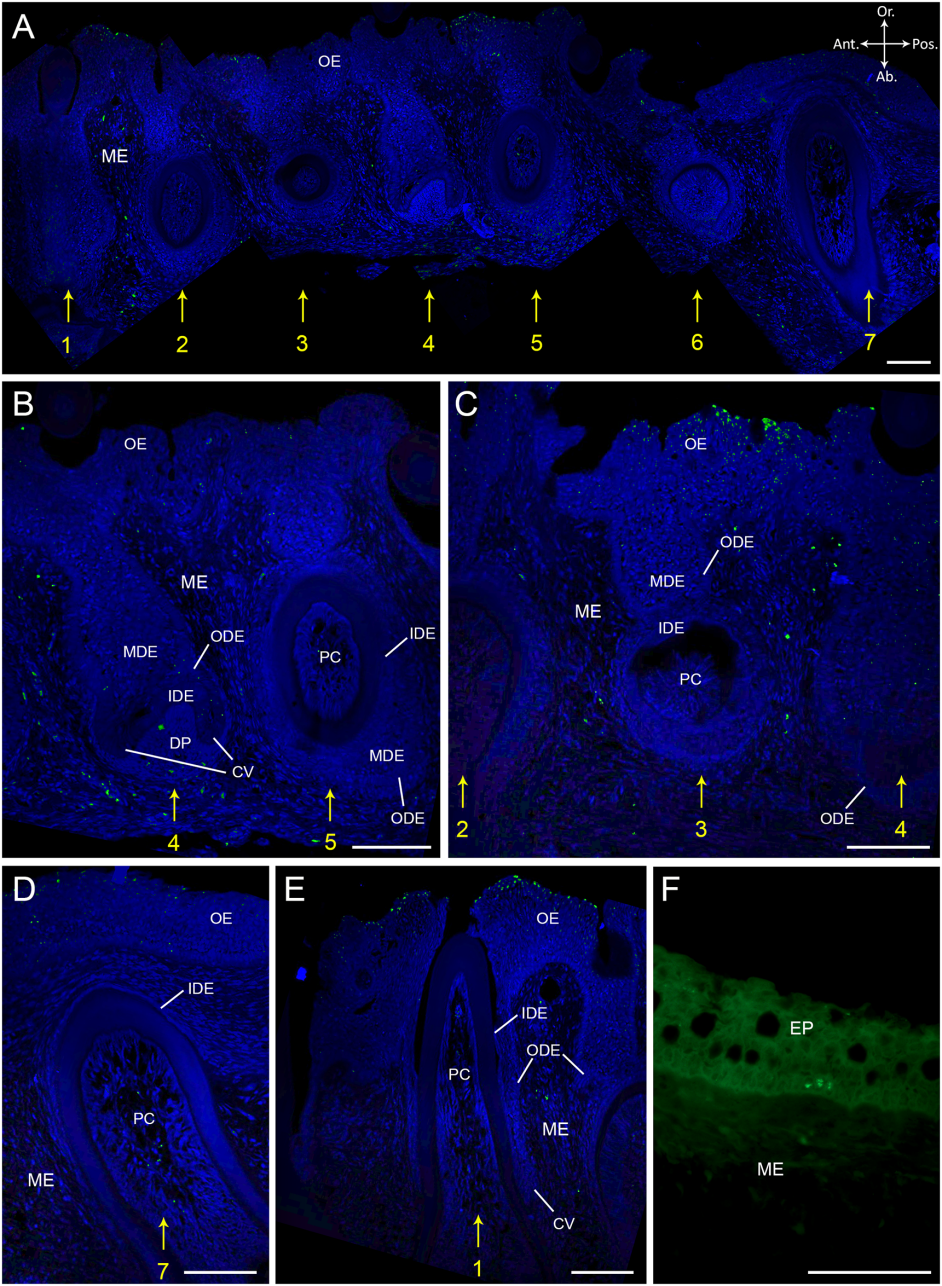
BrdU labelling after eight weeks of chase time in *S*. *salar*. Immunohistological staining of sagittal sections through the lower jaw of *S*. *salar* having experienced a chase time of eight weeks (T4) with BrdU labelled cells in green and DAPI counterstain in blue. (A) shows seven adjacent tooth families in different stages of development. Tooth family one, four and seven have a young functional tooth and a replacement tooth in morphogenesis stage; tooth family two and five have an old tooth in resorption, a young successor, and show initiation of a third tooth germ; tooth family three and six have a mature functional tooth with its successor in cytodifferentiation stage. (B) In tooth family four, nuclei in the cervical loop, ODE and MDE of the successor show small fragments of BrdU label. Bright BrdU label is visible in the mesenchymal cells at the aboral side of the dental papilla. Label is absent in tooth family five. (C) Tooth family three, with a successor in cytodifferentiation stage, shows no BrdU labelled cells. Differentiated cells at the surface of the oral epithelium are brightly BrdU^+^. (D-E) show a young functional tooth of tooth family seven and one, resp., with fragmented BrdU label in the center region of the pulp cavity. (F) Skin epithelium with BrdU^+^ cells (LRCs) in the basal layer of the epithelium. Abbreviations: Ab: aboral; Ant: anterior; CV: cervical loop; DP: dental papilla; EP: skin epithelium; IDE: inner dental epithelium; MDE: middle dental epithelium; ME: mesenchyme; OE: oral epithelium; ODE: outer dental epithelium; Or: oral; PC: pulp cavity; Pos: posterior; asterisk: replacement tooth; yellow numbers indicate tooth family number; scale bars: 50 μm.

In order to determine if label-retaining cells (LRCs) are present in the dental organ of *S*. *salar*, we investigated BrdU labelling in specimens that were subjected to a longer chase time (eight weeks, T4). Seven adjacent tooth families were analyzed. Consistent with the findings of Huysseune et al. (2007) [38], we find recurring patterns of developmental state across these families: (1) tooth family one, four and seven have a young functional tooth and a replacement tooth in morphogenesis stage; (2) tooth family two and five have an old tooth in resorption, a young successor, and display initiation of a third tooth germ, cf. [8]; and finally (3) tooth family three and six have a mature functional tooth with its successor in cytodifferentiation stage (Fig 4A). Thus, similar developmental stages can be found every three tooth loci more posterior or anterior along the jaw.

Where a new tooth germ is initiated, BrdU^+^ cells are completely absent from the dental organ (Fig 4B, tooth family five). However, in a replacement tooth in morphogenesis stage (Fig 4B, tooth family four), some cells in the cervical loop as well as in the ODE close to the cervical loop retain small fragments of label in their nuclei. A few cells, which belong to the MDE and are enclosed by the cervical loop, display a faint and extremely fragmented BrdU label. At the base of the dental papilla and the underlying mesenchyme, BrdU^+^ cells are present, however with fragmented label in their nucleus. In a replacement tooth in late cytodifferentiation, the dental organ is again completely devoid of BrdU^+^ cells (Fig 4C). Yet, the mesenchyme flanking the anterior and posterior end of the dental organ contains BrdU labelled nuclei with non-fragmented label. Finally, the pulp cavity of a young functional tooth (Fig 4D, tooth family seven) has very few BrdU^+^ cells with fragmented label, located in the center. In the oral epithelium, BrdU positive cells are distributed in a fashion similar to what is observed after four weeks of chase time: both fragmented and non-fragmented labelled cells are found in the outermost tier, and a few cells in the basal layer, albeit fragmented (Fig 4C to 4E).

In conclusion, not a single BrdU^+^ cell with intact, i.e. non-fragmented, label could be identified in the dental organ of a tooth family after eight weeks of chase time. In the oral epithelium however, like in the skin (not shown), cells that retained their BrdU label for eight weeks were clearly present (Fig 4F), serving as a positive control for the absence of such cells in the dental organ.

### Long chase time and Sox2 distribution in *Polypterus senegalus* does not support evidence for stem cells in the MDE

**Fig 5:**
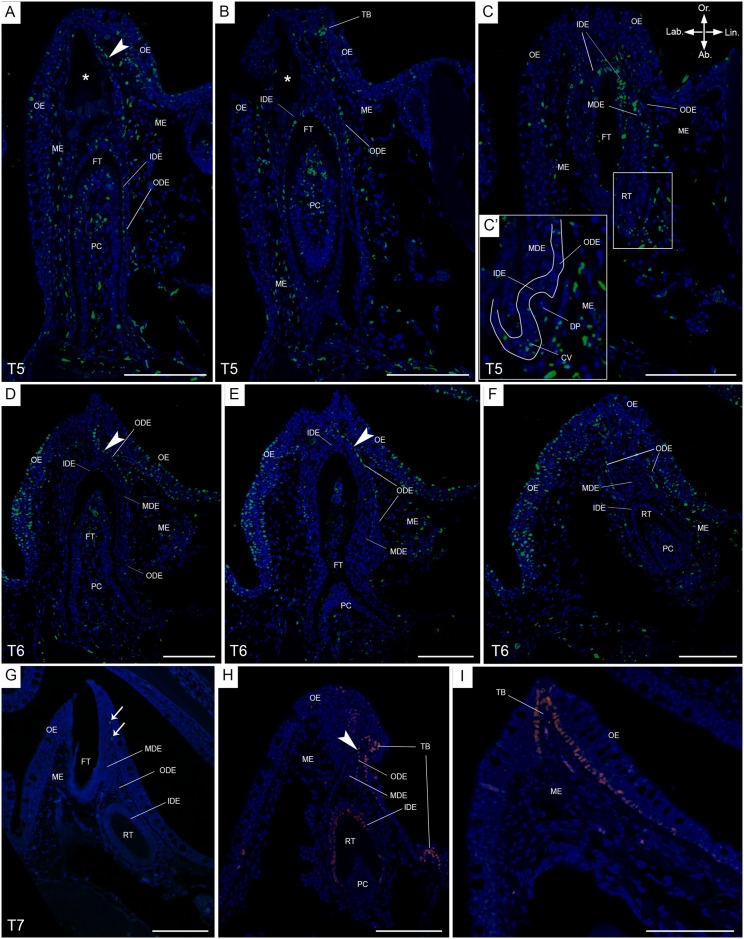
BrdU labelling after long chase times, and Sox2 distribution, in *P*. *senegalus*. Immunohistological staining of transverse sections of a single tooth family on the lower jaw in *P*. *senegalus* after a chase time of six weeks (T5, A-C), eight weeks (T6, D-F) and twelve weeks (T7, G), with BrdU labelled cells in green and DAPI counterstain in blue. In A-C, (A) is the most anterior and (C) the most posterior section. (C’) is a magnification of the secondary successor, in morphogenesis stage. The anterior-posterior axis is perpendicular to the plane of sectioning, with the anterior side pointing upwards. In D-F, (D) is the most anterior and (F) the most posterior section. The anterior-posterior axis is perpendicular to the plane of sectioning, with the anterior side pointing upwards. This tooth family has a mature functional tooth and its successor in an advanced cytodifferentiation stage. (G) BrdU^+^ cells (LRCs) are present in the mesenchyme (arrows). (H-I) Distribution of Sox2 (pink) on transverse sections of a single tooth family (H) and taste bud (I); Sox2^+^ cells are distributed in the ODE transition zone, basal layer of the oral epithelium, IDE of a replacement tooth in cytodifferentiation stage and basal layer of the taste bud. Abbreviations: Ab: aboral; CV: cervical loop; DP: dental papilla; FT: functional tooth; IDE: inner dental epithelium; Lab: labial; Lin: lingual; MDE: middle dental epithelium; ME: mesenchyme; ODE: outer dental epithelium; OE: oral epithelium; Or: oral; PC: pulp cavity; RT: replacement tooth; TB: taste bud; arrowheads: ODE transition zone; asterisk: tooth in resorption; scale bars: 100 μm.

In order to compare the data obtained from *S*. *salar* to another species lacking a successional dental lamina, we applied BrdU labelling in *P*. *senegalus*, and allowed for prolonged chase times (six, eight and twelve weeks, T5-T7, resp., extending the experiments performed in Vandenplas et al. [4]). After six weeks of chase time, BrdU^+^ cells are present in the ODE, known to envelop the complete dental organ, more in particular: (1) in the ODE transition zone, (2) in a young functional tooth close to the cervical loop, and (3) in the cervical loop of a replacement tooth in morphogenesis stage of development (n = 2, Fig 5A to 5C, and data not shown). Importantly, this replacement tooth shows cell nuclei in the cervical loop with a fragmented BrdU label (Fig 5C). Cells with intact BrdU label are absent from the MDE. In the young functional tooth, a gradient of BrdU^+^ cells is visible in the IDE, with few cells with fragmented label close to the cervical loop, many more of such cells more orally, and nuclei with intact label at the very tip (Fig 5A to 5C). Furthermore, BrdU^+^ cells are present in the pulp cavity of a young functional tooth, with intact label in the central region towards the tooth tip, and fragmented label in the periphery, adjoining the tooth matrix (Fig 5A to 5C). BrdU^+^ cells are also found in the mesenchyme surrounding the dental organ, and in the oral epithelium.

Similar to T5, BrdU^+^ cells are absent from the MDE after eight weeks of chase time (T6). The dental organ does not show many BrdU^+^ cells; however, in the ODE transition zone, close to the basal layer of the oral epithelium, BrdU^+^ cells are present with either fragmented or intact label (n = 8, Fig 5D to 5F). The oral epithelium itself has many BrdU labelled cells. These contain non-fragmented label in the outermost layer and both fragmented and non-fragmented label in the intermediate and basal layers (Fig 5D to 5F). The IDE of the replacement tooth in cytodifferentiation shows very few BrdU labelled nuclei, with only fragments of the label remaining (Fig 5F). In contrast with T5, the pulp cavity of the functional tooth no longer shows BrdU^+^ cells in its periphery. Only some cells in the central region at the base and at the tip have retained label (Fig 5D to 5E). The location of BrdU^+^ cells in the pulp cavity of a replacement tooth differs according to developmental stage: they are present in the central region in late cytodifferentiation, but only at the tip region in early cytodifferentitation (Fig 5F, and data not shown). Finally, in the mesenchyme and especially close to the lingual side of the ODE, many cells with intact BrdU label are present.

The longest chase time of our experiment (12 weeks, T7) shows no BrdU^+^ cells in the dental organ or oral epithelium. Only in the mesenchyme in close proximity to the dental organ, occasionally BrdU labelled cells are present (n = 6, Fig 5G, arrow).

Immunohistological staining for Sox2 of *P*. *senegalus* jaws revealed labelled cells in the basal layer of the oral epithelium close to taste buds, in the taste buds themselves, and in ODE transition zone (approximately 10–15 Sox2^+^ cells) (n = 3, Fig 5H to 5I). The latter seamlessly grades into the basal layer of the oral epithelium, which hampers correct interpretation of where the former layer ends and the latter begins. Sox2 distribution in this part of the ODE coincides with areas of BrdU label in chase time T5 and T6. Furthermore, Sox2^+^ cells are absent in the MDE but remarkably occur in the IDE of a successor in late cytodifferentiation stage. Occasional Sox2 labelled cells appear in the mesenchyme and in the oral epithelium away from nearby taste buds. Likewise, immunostaining for Sox2 in *S*. *salar* revealed positive cells restricted to the taste buds and ODE transition zone, but not in the dental organ proper (data not shown).

## Discussion

The 3D reconstruction of the dental organ of a *S*. *salar* tooth family confirms features described earlier in Huysseune and Witten [8] based on histological serial sections, i.e. new tooth germs arising at the lingual and posterior side of the dental organ, the absence of a dental lamina, and the presence of an MDE positioned between the functional tooth and its successor. We used our 3D reconstruction to illustrate the hypothesis regarding the involvement of epithelial stem cells in continuous tooth replacement (Fig 1B). The absence of a dental lamina in *S*. *salar* as described by Huysseune and Witten [8], and confirmed here, adds a mode of tooth formation to that suggested by Reif [17] i.e. dentitions are formed by a dental lamina, a deep epidermal invagination in which successor teeth develop. A dental lamina is also lacking in another salmonid, *O*. *mykiss* [5–7] and in the basal extant osteichthyan *P*. *senegalus* [4]. In these species, successor teeth develop directly from the outer dental epithelium of the predecessor, at the postero-lingual side of the dental organ common to all members of the tooth family. Interestingly, they share the presence of a middle dental epithelium (MDE), an epithelial tier positioned between the inner dental epithelium of the predecessor tooth and the outer dental epithelium of the replacement tooth, and thus restricted to the posterior side of the tooth family. Nonetheless, a MDE-like structure was earlier characterized in *Lepisosteus oculatus* (garfish) as stellate reticulum by Sasagawa and Ishiyama [46]. However, this differs from the MDE as described in *S*. *salar* and *P*. *senegalus* by its large intercellular spaces and reticular aspect [4]. Huysseune and Witten [8], and Vandenplas et al. [4] hypothesized that the mode of tooth replacement without a distinct dental lamina might be ancestral in osteichthyans and that a MDE might functionally substitute for a successional dental lamina.

Here we focused on tooth families in the lower jaw dentition of *S*. *salar* parr stages to (1) determine proliferation in the dental organ, surrounding mesenchyme and oral epithelium, (2) explore the presence of BrdU labelled cells in the different cell layers over increasing chase times, (3) screen for label-retaining cells (LRCs), and (4) finally compare these results with long term BrdU labelling in *P*. *senegalus*. The MDE of *S*. *salar* is located at the postero-lingual side of the functional tooth. After a replacement tooth has been initiated, the MDE shows an extension around the new tooth germ and populates its lingual cervical loop, albeit initially by just a few cells. Meanwhile, the bulk of the MDE, located between the functional tooth and the successor, continues to expand, as indicated in the specimens that received a pulse. Progeny of these cells likely assist in the expansion and reestablishment of the MDE at the postero-lingual side of the tooth family. Whether progeny also translocate to the ODE to eventually contribute to the formation of a new tooth germ, cannot be inferred from our data. Such a scenario was hypothesized earlier for *S*. *salar* [8] and *S*. *canicula* [47] and is similar to what is observed in rodent incisors [34,48]. Thus, together, the MDE and surrounding ODE can be seen as an epithelial population continuously growing in a postero-lingual direction, resembling the dental lamina as described in *O*. *latipes* [13] and. *S*. *canicula* [1,3,47]. Different from the situation in sharks, the bulk of the MDE located between the predecessor and the successor disappears once the latter is advanced in its development. How this is achieved remains elusive. One possibility is that with attachment and eruption of the functional tooth, the MDE is reduced anteriorly, along with IDE and ODE. Whether this is achieved by apoptosis (cf. [49,50]) or is the result of rotation of the functional tooth into position causing integration of these epithelial layers into the general oral epithelium, remains to be determined.

The pulse chase experiment demonstrates the replenishment of the IDE by the cervical loop, contributing cells that will eventually differentiate into enameloid-producing ameloblasts [51]. Likewise odontoblasts appeared to be replenished from the dental papilla. Finally, our data could confirm constant renewal of the oral epithelium from its intensively proliferating basal layer as shown in skin epithelium [52–55].

Our results suggest that the development of early stages in tooth replacement (i.e. initiation and morphogenesis) proceeds swiftly in *S*. *salar*. E.g., in a replacement tooth in morphogenesis stage, there is a decrease to almost disappearance of BrdU labelled cells between T0 and T2 in the dental papilla, which may be explained by one of two scenarios: (1) the initially proliferating mesenchymal cells that replenish the pulp cavity underwent too many divisions for the BrdU label to be detectable after two weeks, or (2) the mesenchymal cells were not condensed and proliferating yet at the moment of BrdU administration, and therefore did not incorporate the BrdU. Both scenarios support the rapid development of this stage. This interpretation is further supported by the rare observations of tooth germs in early initiation, as was previously observed for *P*. *senegalus* [4]. Our data correspond to what is observed in the rainbow trout [23], at least with respect to the stage up to initial calcification. Differences may relate to the size of the fish in the two studies. We suggest that in *S*. *salar*, once the replacement tooth reaches early cytodifferentiation, the process of development slows down until the old functional tooth undergoes resorption, preceded by the initiation of a new tooth germ.

In the stellate reticulum of the mouse incisor, epithelial stem cells are present which divide into (1) a stem cell that replenishes the stem cell niche, and (2) a transient amplifying cell, which translocates to the cervical loop, proliferates and whose descendants finally differentiate into functional ameloblasts [34,35,48]. Rodents are monophyodont and therefore incisors are not replaced but grow continuously. This complicates the comparison between an incisor stellate reticulum and the MDE in *S*. *salar*. The rodent stellate reticulum remains in a fixed position and can act as a stable stem cell niche that is maintained lifelong. In none of the screened tooth families in *S*. *salar* and *P*. *senegalus* could we find LRCs (label-retaining cells) cells in the dental organ after eight weeks chase time. Importantly, LRCs were present in the basal layer of the oral epithelium, consistent with the expected location of epidermal stem cells [56,57]. Furthermore, the LRCs on the surface of the OE could not be considered as slow cycling cells, since this region showed no instantaneous proliferation. They most probably are terminally differentiated cells still present in the OE. However, the observation of intact LRCs in the mesenchyme might suggest the presence of slow cycling cells or terminally differentiated cells. Whether some of these mesenchymal LRCs act as stem cells, involved in tooth cycling, is still largely unknown and definitely needs additional experimentation. Although the absence of LRCs in the dental organs of *S*. *salar* suggests the absence of epithelial stem cells, we cannot exclude their existence based on just this character [13].

Indeed, populations of stem cells that are long-lived yet constantly cycling (and are therefore unable to retain their DNA label) have been identified in hair follicles, intestinal crypts and bone marrow [58]. Populations of quiescent stem cells, which are label-retaining, coexist with adult cycling stem cells [58]. Thus, cells from the MDE with a nuclear label that becomes fragmented over increasing chase times, could still be cycling stem cells. However, until it is shown that cells from the MDE have the potential to generate the epithelial layers of a new tooth germ, e.g. by means of cell lineage tracing, or *in vitro* culture transplantation, their stemness remains elusive. Mechanisms such as budding control by reaction-diffusion similar to what has been described in mouse molars could be considered to regulate repeated tooth formation [59] without necessarily excluding the presence of stem cells. Such a dual mechanism has been demonstrated e.g. in lung development and repair [60,61].

From our study of *P*. *senegalus*, similar conclusions regarding the absence of LRCs can be drawn since no cells with intact label were discovered in the MDE after six (T5), eight (T6) or twelve (T7) weeks of chase time. Furthermore, no Sox2 positive cells were present in the MDE. Remarkably, in *P*. *senegalus* having experienced chase times of six and eight weeks, cells that still carried intact BrdU label (LRCs) were present in the ODE transition zone, that is also Sox2 positive. Using medaka (*O*. *latipes*) Abduweli et al [13] showed LRCs embedded in a Sox2 positive region, and predicted them to be epithelial stem cells involved in generating the new tooth generations. In, e.g., *S*. *canicula* and various species of cichlids, two cellular domains potentially housing stem cells have been hypothesized to be involved in lifelong tooth replacement: (1) a layer intermediate between IDE and ODE, analogous to the stellate reticulum of mouse incisor cervical loops, and (2) the oral epithelium, superficial to each invaginating successional dental lamina [3,62,63]. Yet, the absence, in either *P*. *senegalus* or *S*. *salar*, of transit amplifying cells in the dental organ immediately adjacent to the LRC containing ODE transition zone, strongly argues that these cells do not replenish the dental organ [4]. Thus, co-localisation of *sox2* expression and LRCs is not sufficient evidence to conclude for dental stem cells being involved in tooth turnover, as they must be shown to produce progeny that shift in position as the replacement cycle progresses. Likewise, *S*. *canicula* shows no proliferating cells in the dental lamina where it merges with the oral epithelium [47]. It is nevertheless possible that the LRCs in the ODE transition zone contribute to the renewal of oral epithelium or taste buds in their close proximity.

In conclusion, we have characterized the spatial extent of the middle dental epithelium in the dentition of *S*. *salar*. We have shown that the MDE extends postero-lingually to the newest tooth germ in the tooth family, enclosed by its posterior cervical loop. Together with data from a pulse-chase experiment, these observations suggest that the dental organ grows continuously in a postero-lingual direction. Proliferation in developing replacement teeth occurs not only in the MDE but also in the ODE and cervical loop, thus replenishing the IDE. Widespread proliferation also occurs in the dental papilla prior to final differentiation into odontoblasts. We were unable to find LRCs in the dental organ and therefore our data do not support the hypothesis that stem cells reside in the MDE. Findings obtained for *S*. *salar* are confirmed in *P*. *senegalus*. Finally, in both species we discovered LRCs in the ODE transition zone in long chase times, overlapping with a Sox2 positive region in *Polypterus segenalus*. However, since no replenishment from the surface towards the postero-lingual side of the dental organ could be inferred from the instantaneous pattern of proliferation, it is likely that these cells do not contribute to the formation of the new tooth germ.
